# Soluble Neural-cadherin as a novel biomarker for malignant bone and soft tissue tumors

**DOI:** 10.1186/1471-2407-13-309

**Published:** 2013-06-26

**Authors:** Rui Niimi, Akihiko Matsumine, Takahiro Iino, Shigeto Nakazora, Tomoki Nakamura, Atsumasa Uchida, Akihiro Sudo

**Affiliations:** 1 Department of Orthopaedic Surgery, Mie University Graduate School of Medicine, Tsu, Japan; 2 Mie University, Tsu, Japan

**Keywords:** Sarcoma, Cadherin, Prognosis, Shedding, Biomarker

## Abstract

**Background:**

Neural-cadherin (N-cadherin) is one of the most important molecules involved in tissue morphogenesis, wound healing, and the maintenance of tissue integrity. Recently, the cleavage of N-cadherin has become a focus of attention in the field of cancer biology. Cadherin and their ectodomain proteolytic shedding play important roles during cancer progression. The aims of this study are to investigate the serum soluble N-cadherin (sN-CAD) levels in patients with malignant bone and soft tissue tumors, and to evaluate the prognostic significance of the sN-CAD levels.

**Methods:**

We examined the level of serum sN-CAD using an ELISA in 80 malignant bone and soft tissue tumors (bone sarcoma, n = 23; soft tissue sarcoma, n = 50; metastatic cancer, n = 7) and 87 normal controls. The mean age of the patients was 51 years (range, 10–85 years) and the mean follow-up period was 43 months (range, 1–115 months).

**Results:**

The median serum sN-CAD level was 1,267 ng/ml (range, 135–2,860 ng/ml) in all patients. The mean serum sN-CAD level was 1,269 ng/ml (range, 360–2,860 ng/ml) in sarcoma patients, otherwise 1,246 ng/ml (range, 135–2,140 ng/ml) in cancer patients. The sN-CAD levels in patient were higher than those found in the controls, who had a median serum level of 108 ng/ml (range, 0–540 ng/ml). The patients with tumors larger than 5 cm had higher serum sN-CAD levels than the patients with tumors smaller than 5 cm. The histological grade in the patients with higher serum sN-CAD levels was higher than that in the patients with lower serum sN-CAD levels. A univariate analysis demonstrated that the patients with higher serum sN-CAD levels showed a worse disease-free survival rate, local recurrence-free survival rate, metastasis-free survival rate, and overall survival rate compared to those with lower serum sN-CAD levels. In the multivariate analysis, sN-CAD was an independent factor predicting disease-free survival.

**Conclusions:**

sN-CAD is a biomarker for malignant bone and soft tissue tumors, and a potentially valuable pre-therapeutic prognostic factor in patients with bone and soft tissue sarcoma.

## Background

Musculoskeletal sarcoma is a rare malignancy. Despite the recent advances in treatment for these tumors, the prognosis is still poor. To improve the clinical outcomes of sarcoma patients, the discovery of the mechanisms of tumorigenesis and the identification of early biomarkers for determining the diagnosis/prognosis are required. In particular, the identification of a biomarker that can predict patients at high-risk is important, because such a biomarker could be a useful indicator for determining whether adjuvant therapeutic modalities, such as irradiation and chemotherapy, should be utilized.

The etiology of tumors is multifactorial, and is believed to be the result of inappropriate cell growth, faulty cell differentiation and improper cell–cell and cell–extracellular matrix interactions. In particular, cell-cell adhesion is important in maintaining the tissue architecture. Cadherins are one of the most important proteins involved in cell-cell adhesion. The cadherins constitute a large multigene family of transmembrane glycoproteins that mediate calcium-dependent intercellular adhesion. More than 40 members of the cadherin family have been identified so far [1]. Cytoplasmic domain of the cadherin molecule can form a molecular complex with the catenin family, which link the cadherin to the actin cytoskeleton of the cell. The cadherin-dependent signaling affects fundamental cellular processes such as proliferation, survival, differentiation, cell shape and migration, which in turn influence the tissue morphogenesis and structure. The signaling is also involved in pathogenic events such as carcinogenesis and distant metastasis [2,3]. The loss of cell growth control and architecture disruption is hallmarks of oncogenic transformation. Previous studies have provided evidence that the loss of adhesiveness and increased invasive capacity of tumors cells are associated with a disruption of cell-cell adhesion mediated by malfunction or altered phosphorylation of the cadherin-catenin complex [4-7].

Recent studies showed that N-cadherin can be cleaved by ADAM10 (a disintegrin and metalloproteinase 10). The metalloproteinase domain of the enzyme is responsible for the initial step of N-cadherin processing, which releases soluble active fragments into the extracellular space, and subsequently generates an intracellular C-terminal fragment (CTF) [8,9]. The CTF initiates signaling pathways through the cytoplasmic β-catenin pool. Therefore, the ADAM10-dependent cleavage of N-cadherin modulates cell-cell adhesion, as well as signal transduction.

Recently, many researchers have reported that cadherins and their ectodomain shedding play important roles during cancer progression. A multitude of extracellular proteinases have been identified, and proteolytic shedding of the extracellular domain results in the generation of soluble E-, P- or N-cadherin ectodomains. Elevated levels of circulating soluble E- and P-cadherins have been described in cancer patients compared with healthy controls. For example, Chan et al. showed that the soluble E-cadherin concentration was significantly elevated in 67% of patients with gastric cancer [10]. Soluble N-cadherin (sN-CAD) can be found in the circulation of normal individuals, but is elevated in patients with breast, prostate and bladder carcinoma [11-14]. However, to date, there have been no studies that have investigated the use of the serum sN-CAD levels as a diagnostic or predicting factor.

The aims of this study are to investigate the serum sN-CAD levels in patients with malignant bone and soft tissue tumors, and to evaluate the prognostic significance of the sN-CAD levels.

## Methods

### Patient selection

Serum samples were collected from 87 healthy subjects, 73 bone and soft tissue sarcoma patients, and 7 metastatic cancer patients with musculoskeletal metastases. The details of the clinicopathological features of 73 patients of the bone and soft tissue sarcoma are listed in Table 1. The mean follow-up period was 43 months (range, 1–115 months). The patient group included 36 males and 37 females, with a mean age of 51 years (range, 10–85 years) at the first presentation. There were 23 bone sarcomas, including 14 osteosarcomas (OS), 3 chondrosarcomas, 3 Ewing sarcomas, and 3 chordomas. There were also 50 soft tissue sarcomas, including 10 malignant fibrous histiocytomas (MFH), 9 liposarcomas (5 myxoid liposarcomas, 2 pleomorphic liposarcomas, and 2 dedifferentiated liposarcomas), 6 malignant peripheral nerve sheath tumors (MPNST), 3 synovial sarcomas, 3 dermatofibrosarcoma protuberans, 3 rhabdomyosarcomas, 2 epithelioid sarcomas, 2 clear cell sarcomas, an extraskeletal chondrosarcoma, an extraskeletal osteosarcoma, a myxofibrosarcoma, a malignant granular cell tumor, and 8 unclassifiable spindle cell sarcomas. There were six metastatic cancer to the bone (2 thyroid cancers, 2 renal cancers, 1 lung cancer, and 1 multiple myeloma), and one metastatic cancer to the soft tissue (squamous cell carcinoma). A histological grading of the bone sarcoma was performed according to the grading system proposed by Borders AC [15] and a histological evaluation of soft tissue sarcoma was performed using the grading system of the French Federation of Cancer Centers Sarcoma Group system [16]. The histological grading in soft tissue sarcoma was low grade (grade 1) in 9 sarcomas and in high grade 41 sarcomas (grade 2:20, grade 3:21), while the histological grading in bone sarcoma was low grade in 4 sarcomas and high grade in 19 sarcomas. All patients underwent a complete tumor resection with a wide margin during the initial surgery at our hospital. The diagnoses were primarily based on the morphological appearance based on the results of their reactivity for immunostaining. The 73 samples consisted of 50 primary lesions, 17 local recurrences, and 6 metastases.

**Table 1:** Patient and tumor characteristics

**Factor**	**No. of patients**
	**(N = 73)**
Gender	
Male	36
Female	37
Age (years)	
≦49	32
≧50	41
Size (cm)	
5<	17
5≧	56
Location ^1^	
Upper extremities	7
Lower extremities	39
Trunk	25
Depth	
Superficial	6
Deep	67
Histological grading	
low grade	13
high grade	60
Tumor condition	
Primary	50
Recurrent tumor	17
Metastatic tumor	6
Soluble cadherin	
<1,500 ng/ml	52
≧1,500 ng/ml	21

^1^ two patients with multiple sarcomas were excluded.

At the final follow-up, 26 patients were continuously disease-free, 13 patients had no evidence of disease, 15 patients were alive with disease, and 19 patients died of disease.

The control subjects comprised 28 males and 59 females. The mean age of the control subjects was 46 years (range, 19–89 years). Almost all control subjects younger than 60 years old were healthy volunteers without any medical history of cancer. Those over 60 years of age had conditions such as osteoarthritis, osteoporosis, and so on, and all had C-reactive protein values below 0.5 mg/dl.

This study is in compliance with the principles of the Declaration of Helsinki and written informed consent was obtained from all of the patients included in this study.

### Serum sample collection and storage

Fifty-one serum samples were obtained at open biopsy or initial surgical excision before administration of any chemotherapeutic agent. Seventeen serum samples were obtained at the excision of recurrent tumor. Five serum samples were obtained at the excision of the metastatic lesion. Longitudinal change of the sN-CAD was measured by measuring the serum level at the pre- and post-operative day in a metastatic epitheliod sarcoma patient. Venous blood samples were collected and centrifuged at 2,500 g for 10 min. All sera were stored at -80°C until measurement. All samples were collected under the approval of the ethics committee of the Mie University Graduate School of Medicine.

### Immunoenzymometric assay for the sN-CAD levels

The immunoenzymometric assay for the sN-CAD levels was performed using a home-made ELISA plate, as described in a previous report [17]. All serum samples were diluted 5 times in PBS containing 0.1% bovine serum albumin (BSA). A dilution series of recombinant N-cadherin from 1 to 1,000 ng/ml was prepared (recombinant human N-cadherin/Fc chimera, R&D Systems, Abingdon, UK). A 96 well immunoplate (CN-469949, Nalge Nunc International, Denmark) was coated with 75 μl of the diluted sample overnight at 4°C. The wells were washed with PBS/0.05% Tween-20 and quenched at 37°C with PBS/1% BSA for 1 hr. Next, the plates were washed again (4 times) and incubated with the primary antibody (Mouse GC-4 antibody, Sigma, St. Louis, MO; 1/200 in PBS/0.1% BSA) at 37°C for 2 hr. The plates were then washed again (4 times), and subsequently incubated with a mouse secondary antibody linked to alkaline phosphatase (Goat anti-mouse linked to alkaline phosphatase, Sigma, St. Louis, MO; 1/3,000). The substrate, p-nitrophenylphosphate (N2765, Sigma, St. Louis, MO), was added to the plates, and after 30 min, the optical density of each well was determined with a microplate reader (Molecular Devices, Wokingham, UK) at 405 nm. Measurements were done at least in a triplet for each sample, and the mean value was calculated.

### Statistical analysis

The Mann–Whitney U test was used to analyze the association of the serum sN-CAD levels between healthy subjects and patients. The Mann–Whitney U test was also used to analyze the associations between the serum sN-CAD levels and the clinicopathological variables, such as the type of tumor (bone or soft tissue sarcoma), age, tumor depth, tumor size, histological grade, and the type of malignancy (primary or recurrence/metastasis). We defined the cut-off level of sN-CAD at 1,500 ng/ml to identify the high-risk patient group. The disease-free survival (DFS) was defined as the time from the initial treatment to the date of clinically documented local recurrence/metastasis. The local recurrence-free survival (LRFS) was defined as the time from the initial treatment to the date of clinically documented local recurrence. The metastasis-free survival (MFS) was defined as the time from the initial treatment to the date of clinically documented distant metastasis. The overall survival (OS) was defined as the time from the initial treatment to the date of death from any cause. For the multivariate analysis, a Cox proportional hazards regression model was used to identify the statistically significant differences in the survival and to estimate hazard ratios and 95% confidence intervals. The prognostic variables by the univariate analysis with a p < 0.2 (age, tumor size, histological grading, and sN-CAD) were entered into a Cox multivariate analysis model. A p value < 0.05 was considered to be significant. The analysis was performed using the StatView statistical software package (version 5.0; SAS Institute, Cary, NC, USA). The statistical analysis was performed by N.R. and M.A., and both of whom have responsibility for the results of statistical analysis.

## Results

### Serum sN-CAD levels in sarcoma patients, cancer patients, and control subjects

**Figure 1:**
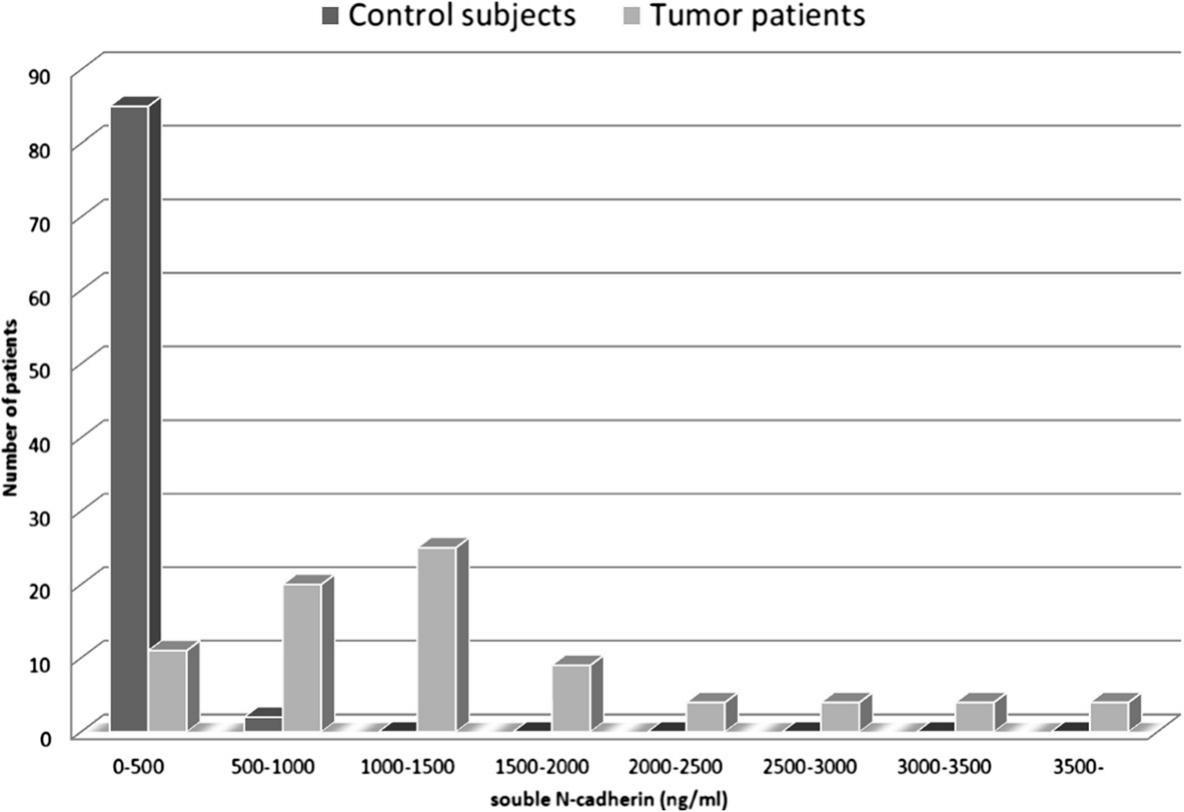
**Serum sN-CAD levels in control subjects and in all patients.** Box plots representing the levels of soluble N-cadherin (sN-CAD) in the sera of 87 control subjects and 73 sarcoma patients. The significance of differences was determined using the Mann–Whitney U test (p <0.001). The levels of sN-CAD in the control subjects were less than 500 ng/ml, while the levels of sN-CAD in the sarcoma patients were distributed over a wide range.

**Figure 2:**
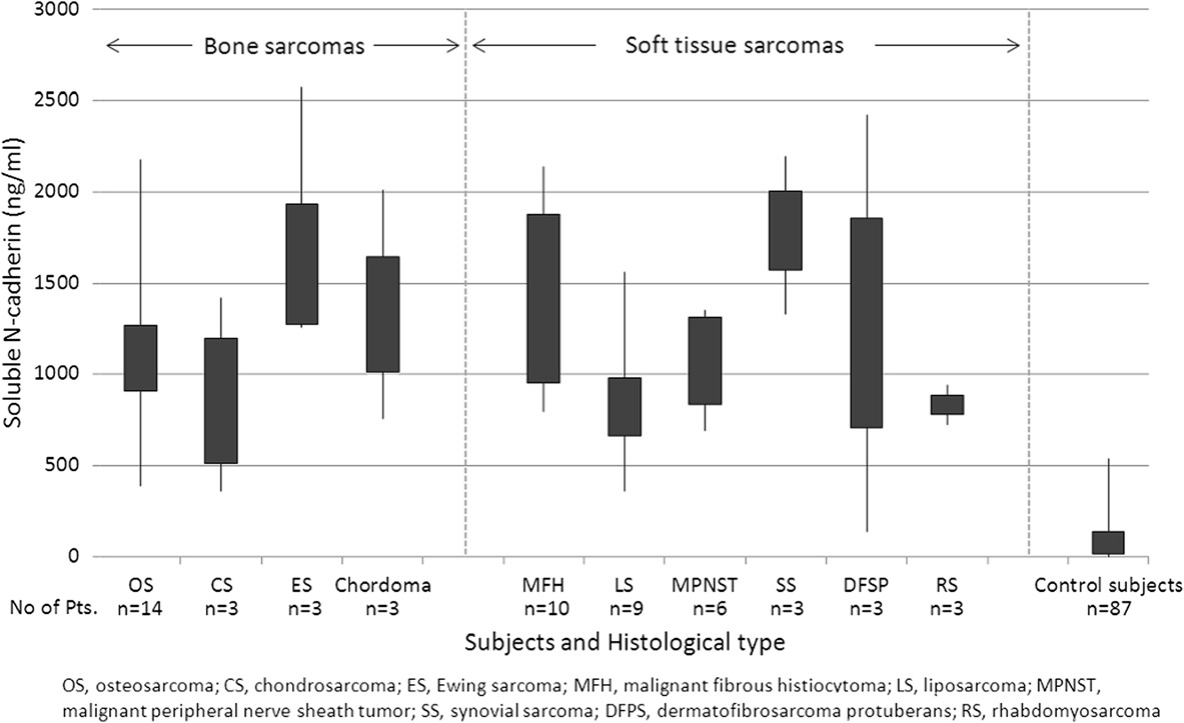
**Distrubution of sN-CAD Levels in sarcoma patient s and in control subjects.** Boxplot showed sN-CAD levels separately for the major bone sarcomas, soft tissue sarcomas and control subjects.

The mean serum sN-CAD level was 1,267 ng/ml (range, 135–2,860 ng/ml) in all patients. The mean serum sN-CAD level was 1,269 ng/ml (range, 360–2,860 ng/ml) in sarcoma patients, otherwise 1,246 ng/ml (range, 135–2,140 ng/ml) in cancer patients with musculoskeletal metastases. The levels measured in the patients were higher than those found in the controls, who had a mean serum level of 108 ng/ml (range, 0–540 ng/ml; p < 0.01, Figure 1). sN-CAD levels in the major sarcoma subgroups and control subjects were indicated in Figure 2. In the controls, levels of sN-CAD under 500 ng/ml were observed, whereas in the sarcoma patients, a wide distribution of sN-CAD levels was observed. Longitudinal change of the sN-CAD was measured in a metastatic epitheliod sarcoma patient. The serum sN-CAD level showed 700 ng/ml just before tumor excision and 525 ng/ml on the next day after surgery.

### Associations between the serum sN-CAD level and the clinicopathological variables

The Mann–Whitney U test was used to analyze the associations between the serum sN-CAD levels and the clinicopathological variables (Table 2). The patients with tumors larger than 5 cm had higher serum sN-CAD levels than the patients with tumors smaller than 5 cm. The histological grade in the patients with higher serum sN-CAD levels was higher than that in the patients with lower serum sN-CAD levels. The levels of serum sN-CAD were not also associated with the type of tumor (bone or soft tissue sarcoma), tumor location, or the condition (primary or recurrence/metastasis) of the lesion.

**Table 2:** **The results of the univariate analysis of the associations between the serum soluble N**-**cadherin levels and the clinicopathological variables**

**Clinicopathological valiables**	**No of patients**	**Soluble N-cadherin (ng/ml)**	***p Value***
Bone tumor	23	1,281 (560–2,575)	0.8914
Soft tissue tumor	50	1,264 (360–2,860)	
Age (years)			0.4665
≦49	32	1,232 (525–2,860)	
≧50	41	1,298 (360–2,580)	
Tumor size ^1^			0.0362
<5 cm	17	1,025 (360–1,900)	
≧5 cm	56	1,343 (360–2,860)	
Depth ^2^			0.5402
Superficial	6	1,364 (390–2,180)	
Deep	67	1,261 (360–2,860)	
Histological grading			0.0482
Low grade	14	1,050 (540–2,575)	
High grade	59	1,321 (360–2,860)	
Primary tumor	51	1,246 (360–2,860)	0.4819
Recurrent / metastatic tumor	22	1,323 (390–2,575)	

^1^ two patients with multiple sarcomas were excluded.

### Prognostic analysis

#### The disease-free survival rate and predictors of events

**Figure 3:**
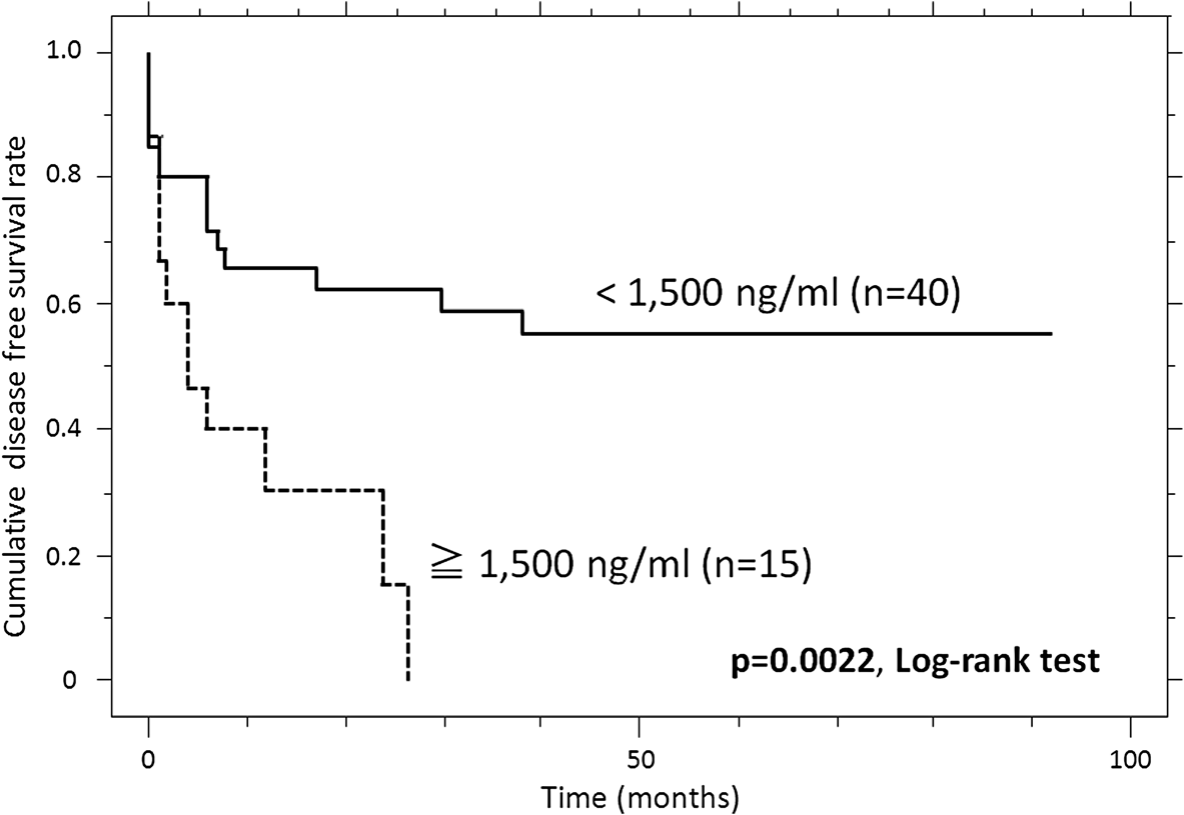
**The cumulative disease**-**free survival (DFS) of patients with high sN-CAD levels, compared to patients with low sN-CAD levels.**

We next compared the OS, DFS, LRFS, and MFS between the patients with high serum sN-CAD levels and the patients with low serum sN-CAD levels. Patients with high sN-CAD levels had a poorer DFS than the patients with low sN-CAD levels (p = 0.0022, Table 3). The estimated DFS at 1, 3 and 5 years was 30.0%, 15.0%, and 0%, respectively, versus 65.7%, 55.1%, and 55.1%, respectively, for patients with high and low sN-CAD levels (Figure 3). A univariate analysis also revealed that there was a significantly poorer outcome for patients with a high tumor histological grade (p = 0.0334, Table 3). The multivariate analysis demonstrated that the sN-CAD levels is an independent prognostic factor (p = 0.0132; Table 4).

**Table 3:** The univariate analyses of the association between patients prognosis and clinicopathological valiables

**Clinicopathological valiables**	**No**	**5-year disease-free survival rate (%)**	***p *Value **^**3**^	**No**	**5-year local recurrence-free survival rate (%)**	***p *Value **^**3**^	**No**	**5-year metastasis-free survival rate (%)**	***p *Value **^**3**^	**No**	**5-year overall survival rate (%)**	***p *Value **^**3**^
Age (years)												
≦49	23	60.9%	0.1603	25	90.0%	0.0621	32	58.9%	0.5899	32	81.8%	0.0517
≧50	32	30.5%		31	62.4%		40	46.1%		41	56.0%	
Gender												
Male	26	50.2%	0.5806	29	76.1%	0.6890	36	60.4%	0.4844	36	68.8%	0.7350
Female	29	35.4%		27	76.5%		36	44.8%		37	66.1%	
Size (cm)												
<5	14	66.1%	0.0616	13	82.5%	0.9879	17	43.5%	0.0159	17	83.3%	0.1327
≧5	41	34.5%		43	73.4%		55	79.5%		56	63.0%	
Depth												
Superficial	3	66.7%	0.8647	3	66.7%	0.6414	6	62.5%	0.6888	6	75.0%	0.7062
Deep	52	42.1%		53	76.9%		66	51.0%		67	66.7%	
Location ^1^												
Trunk	16	34.4%	0.2593	13	65.6%	0.3231	24	54.7%	0.8741	25	69.4%	0.7125
Extremity	37	48.9%		43	77.9%		46	52.7%		46	67.1%	
Histological grading												
Low grade	13	69.2%	0.1171	13	100%	0.1026	14	84.6%	0.0108	14	91.7%	0.0426
High grade	42	31.4%		43	67.2%		58	58.4%		59	60.5%	
Soluble Cadherin ^2^												
Low	40	55.1%	0.0022	40	83.5%	0.0123	51	51.0%	0.0107	52	73.0%	0.0334
High	15	0%		16	60.6%		21	34.9%		21	50.4%	

^1^ two patients with multiple sarcoms were excluded.

^2^ Low means <1,500 ng/ml, and high means ≧1,500 ng/ml.

^3^ Log-rank test.

**Table 4:** **The results of the multivariate analysis of the disease**-**free survival**

**Clinicopathological valiables**	**Relative risk**	**95% confidence interval**	***p value***
Age (years)	2.796	0.608-12.856	0.1865
≦49			
≧50			
Size (cm)	1.474	0.279-7.796	0.6483
<5			
≧5			
Histological grading	0.075	0.005-1.045	0.0540
Low grade			
High grade			
Soluble Cadherin ^1^	6.540	1.480-28.902	0.0132
Low			
High			

^1^ Low means < 1,500 ng/ml, and high means ≧ 1,500 ng/ml.

### Local recurrence-free survival rate and predictors of events

**Figure 4:**
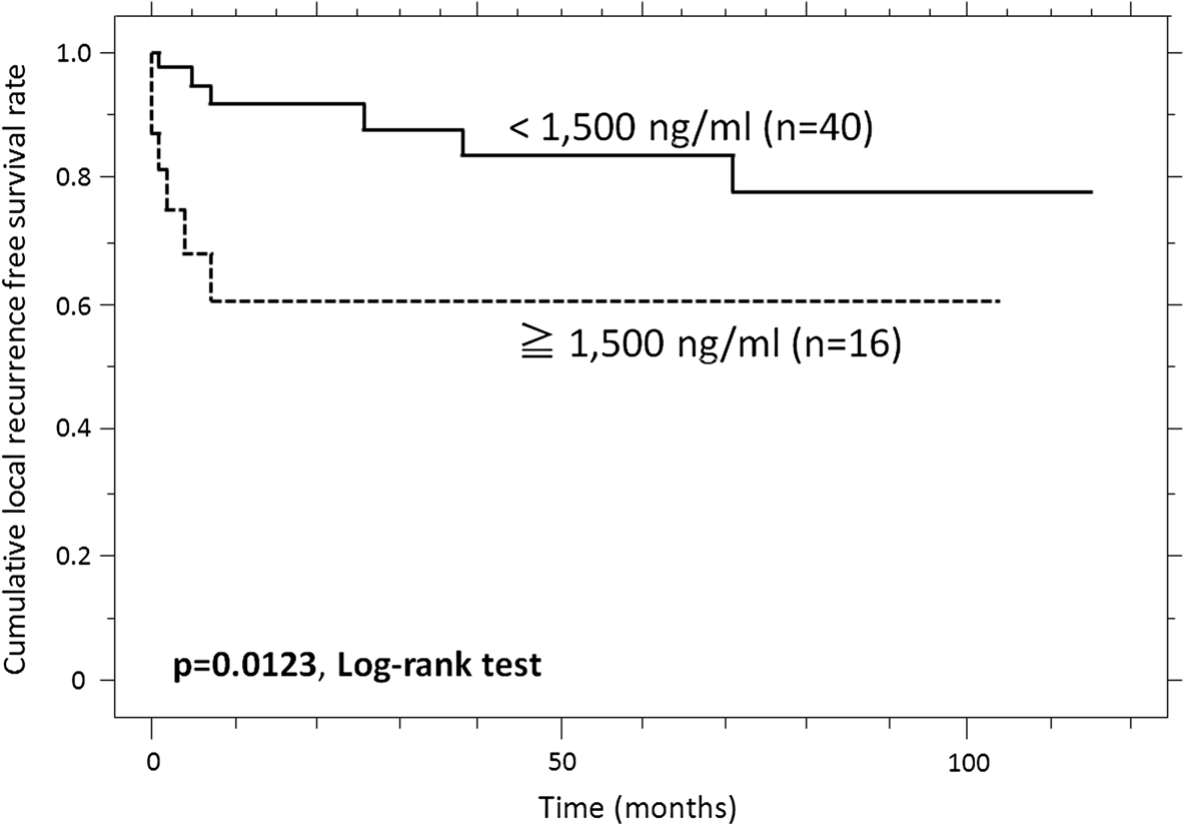
The cumulative local recurrence-free survival (LRFS) of patients with high sN-CAD levels, compared to patients with low sN-CAD levels.

The patients with high sN-CAD levels had a poorer LRFS than the patients with low sN-CAD levels (p = 0.0123, Table 3). The estimated LRFS at 1, 3 and 5 years was 60.6%, 60.6%, and 60.6%, respectively, versus 91.7%, 87.9% and 83.5% respectively, for patients with high and low sN-CAD levels (Figure 4). The high sN-CAD levels lost their prognostic significance in the multivariate analysis (p = 0.1743).

### The metastasis-free survival rate and predictors of events

**Figure 5:**
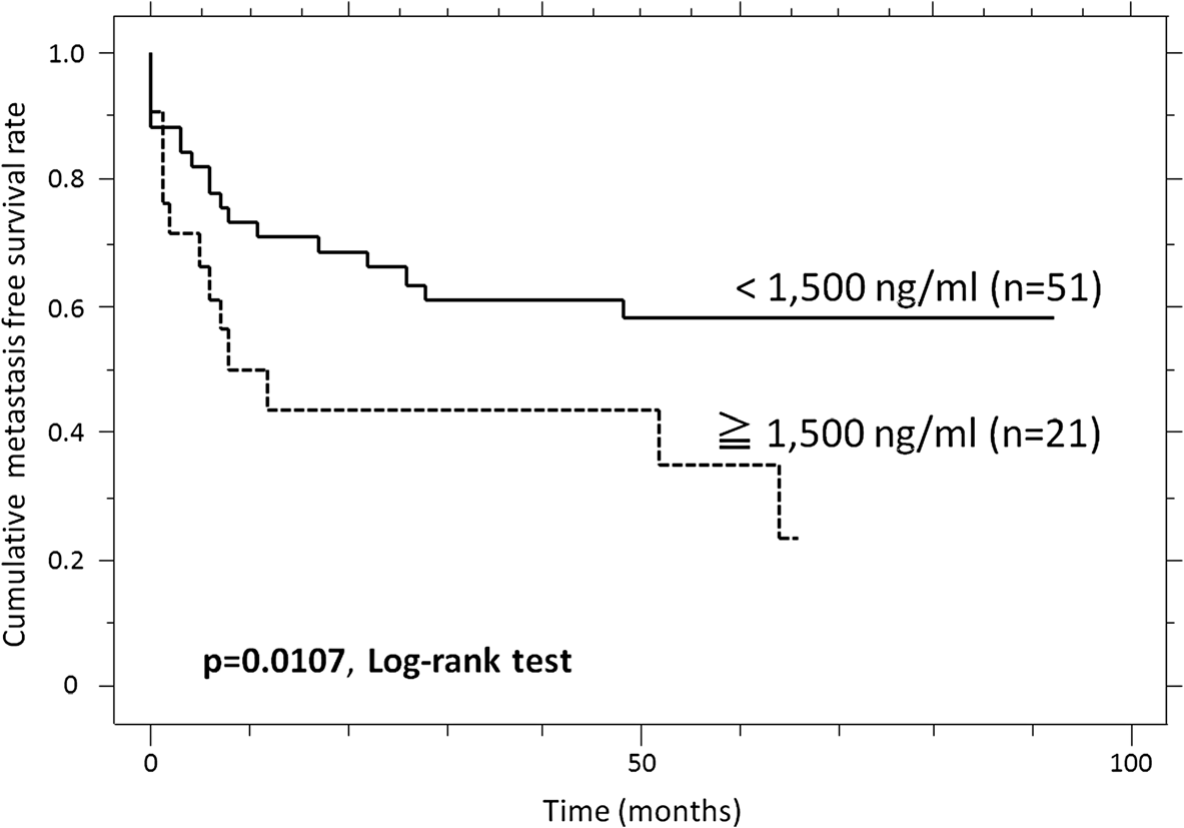
The cumulative metastasis-free survival (MFS) of patients with high sN-CAD levels, compared to patients with low sN-CAD levels.

The patients with high sN-CAD levels had a poorer MFS than the patients with low sN-CAD levels (p = 0.0107, Table 3). The estimated MFS at 1, 3 and 5 years was 43.7%, 43.7%, and 34.9%, respectively, versus 63.1%, 55.4% and 51.0%, respectively, for the high and low sN-CAD levels (Figure 5). A univariate analysis also revealed that there was a significantly poorer outcome for patients with large tumors (p = 0.0159) and high tumor histological grades (p = 0.0108) (Table 3).

### The overall survival rate and predictors of events

**Figure 6:**
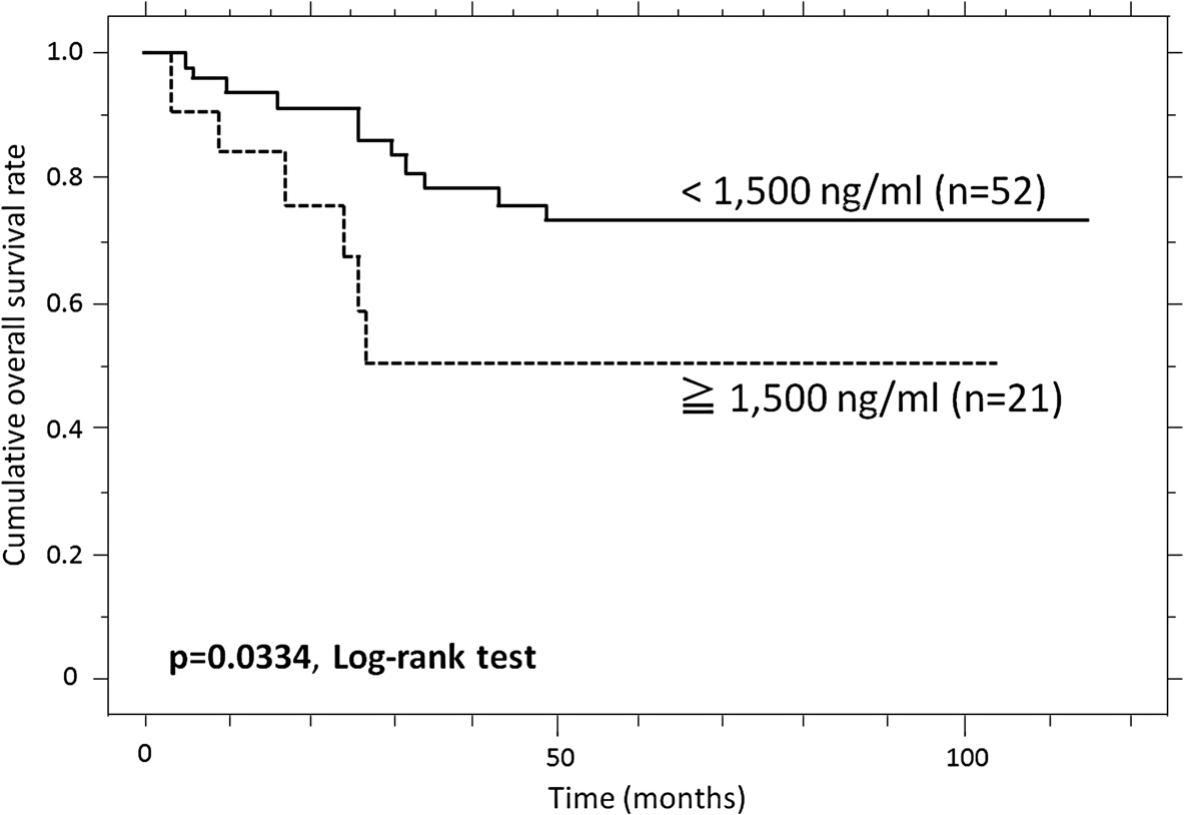
The cumulative overall survival (OS) of patients with high soluble N-cadherin (sN-CAD) levels, compared to patients with low sN-CAD levels.

Patients with high sN-CAD levels had a poorer OS than the patients with low sN-CAD levels (p = 0.0334, Table 3). The estimated OS at 1, 3 and 5 years was 84.0%, 50.4%, and 50.4%, respectively, versus 93.7%, 78.4% and 73.0%, respectively, for patients with high and low sN-CAD levels (Figure 6). A univariate analysis also revealed that there was a significantly poorer outcome for patients with high tumor histological grades (p = 0.004, Table 3). However, both the high sN-CAD levels lost their prognostic significance in the multivariate analysis (Table 5).

**Table 5:** The results of the multivariate analysis of the overall survival

**Clinicopathological valiables**	**Relative risk**	**95% confidence interval**	***p value***
Age (years)	4.087	1.375-12.149	0.0113
≦49			
≧50			
Size (cm)	2.816	0.624-12.706	0.1779
<5			
≧5			
Histological grading	0.135	0.020-0.905	0.0391
Low grade			
High grade			
Soluble Cadherin ^1^	1.858	0.654-5.268	0.2450
Low			
High			

^1^ Low means <1,500 ng/ml, and high means ≧1,500 ng/ml.

## Discussion

In the present study, we found that the serum levels of sN-CAD in patients with malignant bone and soft tissue tumors were higher than those in control subjects. Both the tumor size and the histological grade were positively correlated to the serum sN-CAD levels. The current univariate analyses showed that high serum sN-CAD levels were associated with a decreased OS, DFS, MFS, and LRFS. Multivariate analyses showed an association of a high serum sN-CAD level with a decreased DFS. These results suggest that sN-CAD is a biomarker, and may potentially be valuable as a pre-therapeutic prognosic factor in patients with malignant bone and soft tissue tumors. To the best of our knowledge, this is the first report which indicated the significance of sN-CAD as a biomarker for prognosis in malignant bone and soft tissue tumor patients.

Recently, accumulating evidence has suggested epithelial-mesenchymal transition (EMT) to play a critical role in cancer progression [18]. The EMT is defined by the loss of epithelial characteristics and the acquisition of a mesenchymal phenotype. The cell characteristics are highly affected during the EMT, resulting in altered cell-cell and cell-matrix interactions, and increased cell motility and invasiveness. In epithelial cancers, the EMT is characterized by a switch in cell membrane cadherins (from E- to N-cadherin), a change from apical–basal to front–back polarity and the acquisition of motility, enabled in part by the restructuring of the actin cytoskeleton [19]. The EMT is associated with the invasion and metastasis of various cancers [14,20-23]. Because sarcoma cells have a mesenchymal phenotype, N-cadherin may be one of the key molecules involved in disease progression. However, only a small number of studies about N-cadherin expression in bone and soft tissue sarcoma have been reported. Laskin et al. [24] described that N-cadherin was observed in chordoma (100%), biphasic synovial sarcoma (86%), diffuse mesothelioma (70%), malignant melanoma (56%), epithelioid sarcoma (38%), epithelioid angiosarcoma (25%), poorly differentiated synovial sarcoma (15%), clear cell sarcoma (10%), and monophasic fibrous synovial sarcoma (4%). Kashima et al. [25] found a reduced expression of N-cadherin on the osteosarcoma cell surface, and suggested that the reduced expression of N-cadherin might be due to the proteolytic cleavage of the intact form into the secreted form. However, no study has so far assessed the relationship between the sN-CAD levels and their clinical significance in patients with bone and soft tissue tumors.

The present study showed that the serum sN-CAD levels were correlated with the tumor size (p = 0.02). The several possible causes of the increased levels of sN-CAD in patients with larger tumors can be supposed. First possibility is that the sN-CAD levels may depend on the number of malignant cells. Larger tumor might have a higher sN-CAD level because they are composed of a higher number of cells. Second possibility is that the expression and/or shedding of N-cadherin may increase in rapidly growing tumors. In the current study, the univariate analyses showed that high serum sN-CAD levels were associated with a decreased OS, DFS, MFS, and LRFS. The multivariate analyses showed an association of a high serum sN-CAD level with a decreased DFS. Therefore, our results suggest that a higher sN-CAD level is correlated with the local aggressiveness of the tumor.

In the present study, we confirmed that, poor overall survival was associated with high sN-CAD and high histological grade in the univariate analysis. However, sN-CAD was failed to demonstrate any association with OS in multivariate analysis. The first possible reason is that sN-CAD is not a strong predictor for OS compared with histological grading. The second possible reason is that multivariate analysis couldn’t detect the sN-CAD as a strong predictor for OS, because this study consists of relatively small number of patients. Therefore, we think that the larger scale study is necessary.

Recent studies have demonstrated that N-cadherin can be cleaved by ADAM10, which is one of the ADAM family members [8]. ADAM10 is a transmembrane protein involved in proteolysis and cell adhesion, and has been implicated in the pathogenesis or progression of several cancers, including uterine, ovarian, gastric and colorectal cancer [26]. There have so far been no reports describing the ADAM10 expression in sarcoma tissue, but Matsumura et al. demonstrated that ADAM10 was expressed in a fibrosarcoma cell line [27]. The proteolytic activity of ADAM10 is inhibited by TIMP-1 and 3 [6,8]. Down-regulation of TIMP-1,-3 lead to deregulation of the TIMP-1, -3/ADAM10 pathway, with increased N-cadherin shedding. In fact, Benassi et al. [28] reported the TIMP-1 expression to be either weak or negative in the majority of 53 high grade soft tissue sarcoma samples, and concluded that low levels of negative regulators of proteolysis may be related to the biological aggressiveness of tumors. There was also a report demonstrating a lack of TIMPs expression in almost all high-grade osteosarcomas [29]. Other authors reported that no or minimal expression of TIMP-1 was detected in musculoskeletal sarcomas, and that there was a significant decrease of serum the TIMP-1 levels in sarcoma patients compared to healthy controls [30,31]. Therefore, we believe that the high sN-CAD expression level in high grade sarcoma patients might be the result of a disruption of the balance between the activation and suppression of N-cadherin shedding. Further investigations are therefore warranted.

The chief limitation of this study is the small number of samples examined. As is always the case in studies concerning bone and soft tissue sarcoma, a large number of samples could not be collected due to the rarity of the tumor. This makes it difficult to obtain blood samples from a large number of patients with each histopathology. In addition, bone and soft tissue sarcoma has a variety of pathological classifications, and we were only able to investigate a few samples from each pathological classification. As a result, a statistical analysis could not be performed for each pathological classification due to the small number of samples. The second limitation is that our samples were collected under various conditions, such as from the patients with primary sarcoma, sarcoma with multiple metastases, and so on. To improve the efficacy of using sN-CAD as a biomarker, prospective longitudinal blood collection from numerous patients, for example, collection of blood preoperatively, ximmediately after surgery, and at the time when sarcoma recurs, is indispensible to improve the reliability of sN-CAD as a biomarker. The third limitation is concerning the cut-off value. In this study, we determined the cut-off value in consideration of mean sN-CAD and clinical impact. As described above, a large number of samples could not be collected due to the rarity of the tumor and adequate cut-off value is not established. Further investigation is warrant.

Despite these limitations, we believe that this study is of great value for the diagnosis and treatment of these tumors. Clinically, tumor factors such as tumor-node-metastasis staging, differentiation, histological classification, and the tumor size are known to have strong prognostic value. However, a prognostic indicator that could predict the operability, survival rate, and recurrence rate before the pathology of a resected specimen is available (and, hence, before surgery) would be particularly helpful. This would provide important guidance and clues for the selection of an optimal therapeutic approach. In this study, we identified that the pre-therapeutic level of sN-CAD could serve such a purpose, and could be determined by using a simple enzyme-linked immunosorbent assay.

## Conclusion

In conclusion, our results indicate that sN-CAD is present in significantly higher amounts in patients with malignant bone and soft tissue tumors than in healthy subjects. In addition, a high serum sN-CAD level is associated with a poor outcome in musculoskeletal tumor patients. sN-CAD is therefore a potentially valuable pretherapeutic factor for predicting the long-term survival in patients. Further studies involving a larger sample and longer follow-up are necessary to verify these results.

## Abbreviations

N-cadherin: Neural-cadherin; sN-CAD: Soluble N-cadherin; ADAM10: A disintegrin and metalloproteinase 10; CTF: C-terminal fragment; OS: Osteosarcomas; MFH: Malignant fibrous histiocytomas; MPNST: Malignant peripheral nerve sheath tumors; PBS: Phosphate buffered saline; BSA: Bovine serum albumin; HRP: Horseradish peroxidase; DFS: Disease-free survival; LRFS: Local recurrence-free survival; MFS: Metastasis-free survival; OS: Overall survival; EMT: Epithelial-mesenchymal transition; TIMP: Tissue inhibitor of metalloproteinase.

## Competing interests

The authors declare that they have no competing interests.

## Authors’ contributions

RN carried out plasma collection, statistical analysis, and drafted the manuscript. AM participated in study design, statistical analysis, and draft the manuscript. IT helped with the experiments. SN helped with the experiments. TN carried out analysis. AU directed the research project. AS drafted the manuscript and gave final approval of the manuscript. All authors read and approved the final manuscript.

## Pre-publication history

The pre-publication history for this paper can be accessed here:

http://www.biomedcentral.com/1471-2407/13/309/prepub
